# The Mitochondrial Genomes of Neuropteridan Insects and Implications for the Phylogeny of Neuroptera

**DOI:** 10.3390/genes10020108

**Published:** 2019-02-01

**Authors:** Nan Song, Xin-Xin Li, Qing Zhai, Hakan Bozdoğan, Xin-Ming Yin

**Affiliations:** 1 College of Plant Protection, Henan Agricultural University, Zhengzhou 450002, China; lixinxin412@126.com (X.-X.L.); zhaiqing@henau.edu.cn (Q.Z.); 2 Department of Plant and Animal Production, Kirsehir Vocational School of Technical Sciences, Ahi Evran University, 40100 Kirsehir, Turkey; hakan.bozdogan@ahievran.edu.tr

**Keywords:** mitochondrial genome, phylogeny, Neuroptera, Myrmeleontidae

## Abstract

The higher-level phylogeny of Neuroptera is explored here based on the newly determined mitochondrial genomic data, with a special focus on the interfamilial relationships of this group. Despite considerable progress in our understanding of neuropteran relationships, several mutually exclusive hypotheses have come out according to morphology-based analyses and molecular sequence data. The evaluation of these hypotheses is hampered by the limited taxonomic coverage of previous studies. In this paper, we sequenced four mitochondrial genomes to improve the taxonomic sampling for families: Myrmeleontidae, Ascalaphidae and outgroup Corydalidae. Phylogenetic analyses were run using various inference methods to (1) confirm that Coniopterygidae is sister to all other Neuroptera; (2) place Hemerobiidae as sister to Chrysopidae; (3) support the monophyly of Myrmeleontiformia and define its interfamilial relationships; and (4) recover Myrmeleontidae as paraphyletic due to the nested Ascalaphidae.

## 1. Introduction

Neuropterida is an early-diverging lineage of holometabolous insects with nearly 6500 described species classified into 20 families [1]. This group is generally recognized as an insect superorder, consisting of the orders Neuroptera (lacewings, mantidflies, antlions and their relatives), Megaloptera (alderflies, dobsonflies and fishflies) and Raphidioptera (snakeflies). Neuroptera is a relatively large clade within Neuropterida, including about 6000 species in 15~17 families [2,3,4]. The monophyly of Neuroptera has been well supported by morphological and molecular data [2,4,5], although its internal relationships are still under debate [3].

Based on the morphological characters, the taxon Neuroptera is divided into three suborders Nevrorthiformia, Myrmeleontiformia and Hemerobiiformia [2,6,7]. Antlions (Myrmeleontiformia: Myrmeleontidae) are composed of over 1630 species [8] and form the most diverse family within Neuropterida. The antlions are well known for their remarkable pit building behavior. Larvae of some species are able to construct pitfall traps in loose material to catch ants or other prey. Moreover, larvae of antlions exhibit distinguished morphological characteristics [9], such as the complex piercing and sucking mouthparts. Determining the phylogenetic relationship of antlions to other neuropteran insects is a critical step toward categorizing their biological diversity. A cladistic analysis based on morphological characters indicated a sister-group relationship between Myrmeleontidae and Ascalaphidae [2]. This hypothesis is further corroborated by molecular studies based on multiple genes [10] and mitochondrial genome sequences [11,12,13]. However, some analyses demonstrated the paraphyly of Myrmeleontidae, with regard to Ascalaphidae [4,5,14]. 

Mitochondrial genomes have been widely utilized for insect phylogeny reconstruction [5,11,12,13,15,16,17,18,19,20,21,22,23]. Recent studies have demonstrated that mitochondrial genome sequences are useful for resolving the higher-level relationships within Neuropterida [5,11,12,13]. As of November 2018, 45 complete or nearly complete neuropteran mitogenomes from sixteen families are available on GenBank. Although these molecular data contributed to the resolution of neuropteran relationships in recent studies [11,12,13], the number of sequenced species was still limited in comparison to the total number of known Neuroptera species. In this paper, we determined mitochondrial genome sequences for two species from the family Myrmeleontidae (Neuroptera), one from both Ascalaphidae (Neuroptera) and Corydalidae (Megaloptera), using next-generation sequencing approach, to improve taxonomic sampling for the reconstruction of phylogenetic relationships within Neuroptera. 

In the phylogenetic analyses of combined mtDNA gene sequences, some species from both outgroup (i.e., *Xanthostigma gobicola* in Raphidioptera) and ingroup (i.e., four representatives of Coniopterygidae and Dilaridae in Neuroptera) displayed the obviously long branch lengths. The maximum likelihood analyses using the site-homogeneous GTR model resulted in a non-monophyletic Neuroptera, due to the outside position of Coniopterygidae. The potential long-branch attraction between Raphidioptera and Coniopterygidae may lead to unexpected result. Sequence comparative analyses showed that current mitogenomic data was affected by both nucleotide compositional heterogeneity and rate heterogeneity, because the sequences leading to the long-branched taxa were more heterogeneous than others and had the higher nonsynonymous substitution rate. Recent studies have suggested that the site-heterogeneous CAT-GTR model [24] implemented in PhyloBayes [25,26] can reduce the systematic errors [27,28] and improved the accuracy of phylogenetic reconstruction using mitogenomic data [5,17,18,19,22,23,29]. The site-heterogeneous CAT-GTR model can better account for data heterogeneities by the Dirichlet processes [24,25]. Different categories of substitution processes are estimated for sequence sites and the global exchange rates can be inferred from the data [25]. The site-heterogeneous CAT-GTR model is thus more effective in handling LBA artifacts than the site-homogeneous model.

The aim of this study is to investigate the interfamilial relationships of Neuroptera by using the expanded mitochondrial genome sequence data, with an emphasis on the affinity of Myrmeleontidae with Ascalaphidae. The site-heterogeneous CAT-GTR model recovered the monophyly of Neuroptera and supported the Myrmeleontidae as a paraphyletic group with respect to Ascalaphidae.

## 2. Materials and Methods

### 2.1. Taxonomic Sampling

Taxa examined here included 49 neuropteran taxa which represented 16 families [3,4] recognized in this order (Table 1). This study also included available mitochondrial genomes of three species from Megaloptera and of three species from Raphidioptera for outgroup comparison [4,5,13]. 

Samples of two species from Myrmeleontidae (*Dendroleon pantherinus* and *Euroleon nostras*), of one species from both Ascalaphidae (*Suhpalacsa* sp.) and Corydalidae (*Neochauliodes punctatolosus*) were collected from Three-lake in Guangshui, Hubei, China. The specimens were preserved in 95% ethanol and stored at −20 ℃ for further sequencing. Voucher specimens have been deposited at the Entomological Museum of Henan Agricultural University.

### 2.2. Molecular Data Collection

Total genomic DNA was extracted from the thoracic muscle of individual specimen using the TIANamp Micro DNA Kit (TIANGEN BIOTECH CO., LTD, Beijing, China), following manufacturers’ instructions. The DNA concentration was measured by Nucleic acid-protein analyzer (QUAWELL TECHNOLOGY INC., Sunnyvale, CA, United States).

Genomic DNA for each sample was added into four different libraries, respectively. Besides the species determined in this study, each library also contained 20 unrelated samples, which are the insect species from the Hemiptera or Coleoptera. Equimolar amounts of genomic DNA from each sample were mixed into the library to improve the sequencing efficiency of each species. Libraries were prepared by using the Illumina TruSeqTM DNA Sample Prep Kit (Illumina, San Diego, CA, USA), with the insert size of 350 bp. The subsequent sequencing was conducted in a single lane with 150 bp PE reads on an Illumina HiSeq X Ten platform (Beijing Novogene Bioinformatics Technology Co., Ltd, Beijing, China). 

Raw reads were filtered with NGS QC Toolkit using default settings [30]. Reads containing adapters and poly-N and low-quality reads were removed from raw data. At the same time, phred quality scores of 20 and 30 (i.e., Q20 means that the chances of this base being called incorrectly are 1 in 100 and Q30 means 1 in 1000), GC-content and sequence duplication level of the cleaned data were calculated. All the downstream analyses were based on clean data with high quality (avg. Q20 > 90% and avg. Q30 > 80%). *De novo* assembly for data from pooled sequencing was performed with IDBA-UD v. 1.1.1 [31]. The assemblies were constructed using 200 for the setting of the minimum size of a contig and an initial k-mer size of 40, an iteration size of 10 and a maximum k-mer size of 90.

The mitogenomic assemblies were identified by blasting with the prior mitochondrial baiting sequences (*cox1*-5’) of four neuropteridan species newly sequenced in this study, which were amplified by standard PCR reactions with the primers designed by Song et al. (2016) [22]. The mitochondrial genome annotations were conducted using the MITOS server [32], under default settings and the invertebrate genetic code for the mitochondrial DNA. The gene boundaries were further refined by alignment with homologous sequences of neuropteridan species (see Table 1 for details). To check the quality of the assembled mtDNA sequences, the mappings to the mitochondrial contigs were conducted using BWA v. 0.7.5 [33]. Alignments produced in SAM format were converted to sorted BAM format by SAMtools v. 0.1.19 [34]. Statistics for nucleotide coverage were generated with Qualimap v.2.2.1 [35]. 

### 2.3. Sequence Alignment

DNA sequences were aligned separately for each protein-coding gene in TranslatorX [36], with the invertebrate genetic code and using MAFFT [37] for the amino-acid alignment. At the same time, Gblocks v.0.91b [38] was applied to remove the ambiguously aligned positions, with all options for a less stringent selection. RNA genes were separately aligned using MAFFT under the iterative refinement method incorporating the most accurate local pairwise alignment information (E-INS-i) and ambiguously aligned sites were pruned using Gblocks with the same settings mentioned above. Gaps in the alignments were stripped by Gap Strip/Squeeze v2.1.0, with 40% Gap tolerance (http://www.hiv.lanl.gov/content/sequence/GAPSTREEZE/gap.html). The alignments were concatenated using FASconCAT_v1.0 [39]. 

We created three data sets with varying gene content under the full 55 taxa for the preliminary phylogenetic analyses: (1) PCGRNA (14,535 bp): the nucleotide data with all genes (protein-coding, ribosomal RNA and transfer RNA genes); (2) PCGRNA_Gblocks (13,925 bp): all gene alignments masked by Gblocks were concatenated; (3) PCG12RNA (10,755 bp): the 13 protein-coding genes excluding the third codon position were combined with RNA gene alignments with Gblocks treatment. In addition, a taxon reduced data set of 52taxa_PCGRNA (14,535 bp), excluding the long-branched Raphidioptera, was compiled to investigate the potential long-branch attraction effect.

### 2.4. DNA Analyses

The heterogeneity of sequence divergences within the alignments was analyzed by using AliGROOVE procedure [40], with the default sliding window size and the gaps being treated as ambiguous characters. Nucleotide substitution saturation was assessed for each codon position of the protein-coding genes, the whole protein-coding gene alignment, tRNA genes, rRNA genes and the concatenated data set of tRNA and rRNA genes, by using Xia’s method [41,42] as implemented in DAMBE 5 [43]. We ran the program yn00 from the package PAML [44] to calculate the nonsynonymous (*dN*) and synonymous (*dS*) substitution rates of protein-coding genes, with the method of Yang and Nielsen [45]. 

### 2.5. Phylogenetic Analyses

Phylogenetic analyses of the three combined data sets mentioned above were conducted using maximum likelihood (ML) and Bayesian inference (BI) approaches. Partitioned ML analyses were performed using the IQ-TREE [46] as implemented on the web server (http://iqtree.cibiv.univie.ac.at/). The data blocks were defined by gene and by codon for the 13 protein-coding genes without data treatments, while the data blocks were defined by gene only for the data alignments masked by Gblocks or with the third codon position excluded. The 22 tRNA genes and two rRNA genes were considered as two separate partitions. The data set of PCGRNA contained 41 partitions, while the PCGRNA_Gblocks and the PCG12RNA included 15 partitions, respectively. The best-fit substitution models were determined (Supplementary Table S1) using the Auto function on W-IQ-TREE with ModelFinder [47]. We performed 10,000 ultrafast [48] bootstrap replicates to investigate nodal support across the topology.

Bayesian inference (BI) was performed using PhyloBayes MPI [24,26] as implemented in the CIPRES Science Gateway [49]. Two independent runs were performed for each analysis and each run implemented two Markov chain Monte Carlo (MCMC) chains in parallel for at least 30,000 iterations. The CAT-GTR model was applied to Bayesian tree searches on nucleotide data sets. The values of maxdiff (maximum split frequency) < 0.1 and ESS (effective sample size) > 100 were acknowledged as a good indicator of convergence. The first 1000 trees of each MCMC were treated as the burn-in and the majority-rule consensus tree was computed from the remaining trees of the two chains of each Bayesian inference analysis.

## 3. Results

### 3.1. Reconstruction of Mitochondrial Genomes

As shown in Table 2, at least 128 million high-quality PE reads were produced for each library. Most of these clean reads are non-mitochondrial sequences, because only < 0.3% reads can match with the reference mitochondrial contigs assembled by IDBA-UD. Sequencing depth did not correlate with the completeness of mitochondrial contigs. A relatively complete mitochondrial genome sequence was identified in a single large contig for three species, namely *D. pantherinus*, *E. nostras* and *Suhpalacsa* sp. The mitochondrial genome sequence of *N. punctatolosus* was assembled from two separate contigs. The average depth of coverage of mitochondrial genome sequences was generally high, ranging from 49 to 538.

### 3.2. Genome Structure

**Figure 1:**
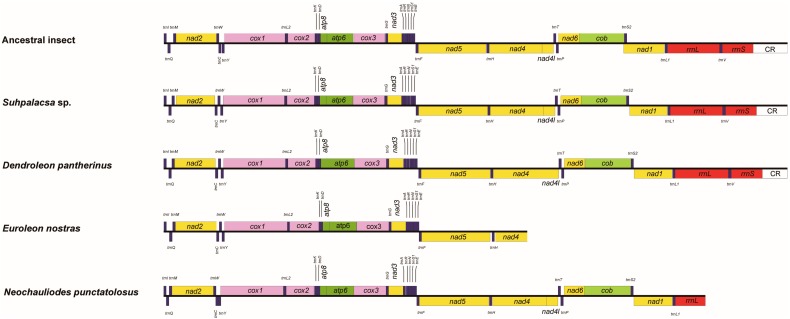
Comparison of structures of mitochondrial genomes among *Suhpalacsa* sp., *Dendroleon pantherinus*, *Euroleon nostras*, *Neochauliodes punctatolosus* and the putative ancestral insect. Linearized representation illustrates the gene arrangements of the circular genomes. The mitochondrial genomes of *Suhpalacsa* sp. and *D. pantherinus* are nearly complete. The *E. nostras* and *N. punctatolosus* have the partial mitochondrial genomes. The transcriptional direction of mitochondrial genes labeled above the line is from left to right and those labeled below the line indicate the opposite direction.

The mitochondrial genome sequences of *Suhpalacsa* sp. and *D. pantherinus* were 15,540 bp and 15,416 bp in size, respectively. These two mitochondrial genomes included a typical set of 37 genes and a partial control region. The mitochondrial genome sequences of *N. punctatolosus* and *E. nostras* were 13,404 bp and 9095 bp in size, which consisted of 35 and 26 mitochondrial genes (Figure 1), respectively. Besides the control region, there was a long non-coding region (54–73 bp) present downstream of *trnI* in the major strand in three neuropteran mitochondrial genomes (i.e., *D. pantherinus*, *E. nostras* and *Suhpalacsa* sp.). The mitochondrial genome of *D. pantherinus* also contained two intergenic regions with 49 bp and 58 bp, which were located between *trnE* and *trnF* and between *nad1* and *trnL*, respectively. Other intergenic identified regions were no more than 10 bp length. No remarkable overlapping genes were found. 

The gene order of *trnC*-*trnW*-*trnY* was observed in the newly sequenced mitochondrial genomes for *Suhpalacsa* sp., *D. pantherinus* and *E. nostras*, while the mitochondrial genome of *N. punctatolosus* had a different arrangement (*trnW*-*trnC*-*trnY*) (Figure 1). The secondary structures of the full set of tRNA genes for *D. pantherinus* and *Suhpalacsa* sp. were shown in Figure S1. The length of the tRNA genes ranges from 61 bp to 71 bp. All tRNA genes exhibited the typical cloverleaf structure, except for *trnS1(AGN)*. A single loop structure instead of the stem-and-loop structure formed the DHU arm of *trnS(AGN)*.

**Figure 2:**
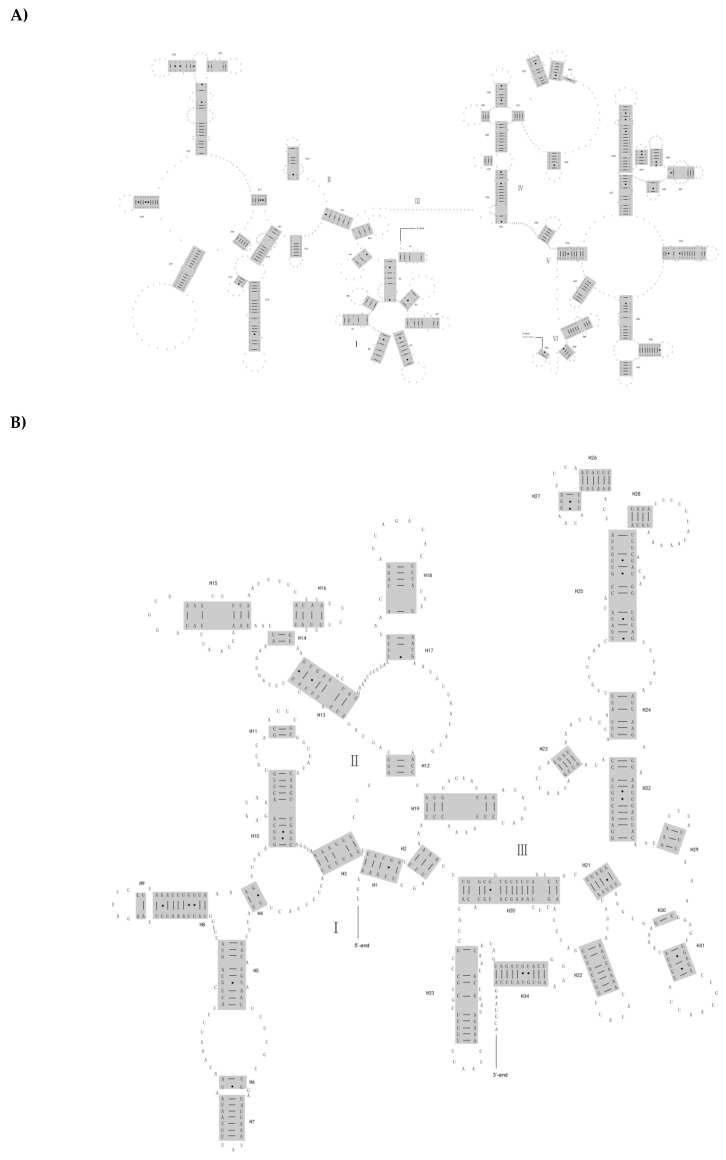
The secondary structures of (**A**) *rrnL* and (**B**) *rrnS* predicted for *Dendroleon pantherinus*.

For the two nearly complete mitochondrial genomes of *D. pantherinus* and *Suhpalacsa* sp., the *rrnL* and *rrnS* were respectively identified between *trnL1* and *trnV* and between *trnV* and control region, with the lengths of 1324 bp and 1315 bp and 775 bp and 778 bp. The gene location and gene lengths were similar to other published neuropteran insects [5,11,12]. Mitochondrial genomes of other two species sequenced (i.e., *E. nostras* and *N. punctatolosus*) are partial and lack the complete rRNA genes (Figure 1). The secondary structures of rRNA genes were inferred with reference to models proposed in the study of Yan et al. (2014) [11]. Figure 2 and Figure S2 showed the secondary structures for *rrnL* and *rrnS* of *D. pantherinus* and *Suhpalacsa* sp., respectively. The *rrnL* consisted of I, II, IV, V and VI structural domains and 50 helices. The domain III was missing because the sequence region between domain II and IV was too short to form the corresponding stem and/or loop structures. There were 275 canonical pairings (A-U and G-C) identified in the *rrnL* secondary structures of *D. pantherinus* and *Suhpalacsa* sp.. The number of non-canonical pairings for two species was 70 (G-U: 47, U-U: 13, A-A: 4, A-G: 4, A-C: 1, U-C: 1) and 73 (G-U: 50, U-U: 18, A-A: 1, A-G: 2, A-C: 1, U-C: 1), which were similar to those observed in previous study [11]. The predicted secondary structure of the mitochondrial *rrnS* gene contained four typical domains (I-IV) and 34 helices. The *rrnS* secondary structures of *D. pantherinus* and *Suhpalacsa* sp. had 161 and 163 canonical pairings, respectively. The number of non-canonical pairings was 41 (G-U: 27, U-U: 9, A-A: 1, A-G: 1, A-C: 2, G-G: 1) and 46 (G-U: 23, U-U: 16, A-A: 1, A-G: 5, A-C: 1).

### 3.3. Characteristics of Data Matrix

**Figure 3:**
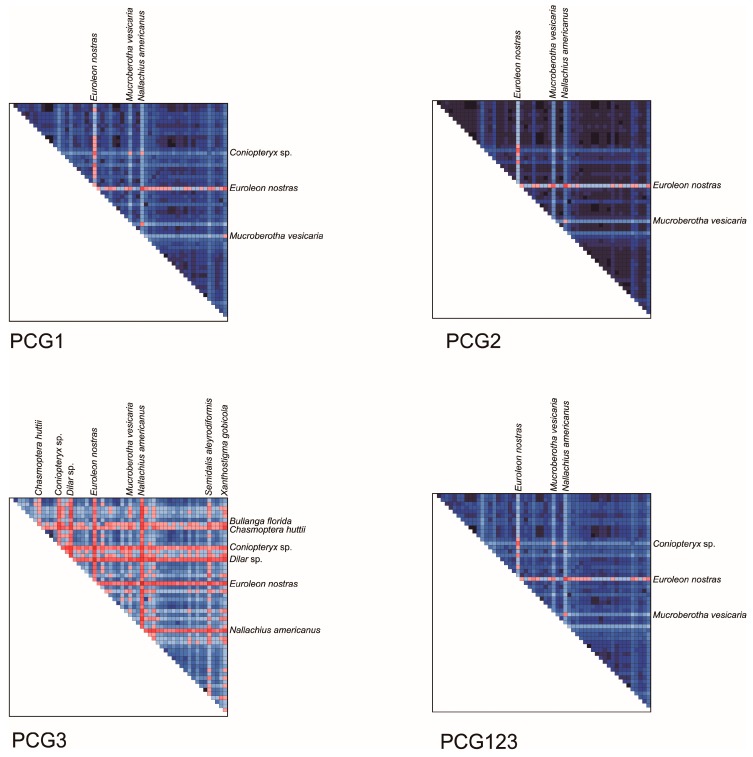
AliGROOVE heat maps of pairwise sequence comparisons for the protein coding genes with 55 taxa. The AliGROOVE graph shows the mean similarity scores between sequences. AliGROOVE scores range from −1 (indicating great difference in rates from the remainder of the data set, i.e., red coloring implies the significant heterogeneity) to +1 (indicating rates match all other comparisons, i.e., blue labeling).

The results of base compositional heterogeneity analyses showed both the first codon positions and the second codon positions had low heterogeneity of sequence composition. In contrast, a remarkably higher heterogeneity was found in the third codon positions of protein-coding genes (Figure 3). The mitochondrial genome sequences of *Semidalis aleyrodiformis*, *Coniopteryx* sp., *Nallachius americanus*, *Dilar* sp. and *E. nostras* were more heterogeneous than other neuropteridan species. Data masked by Gblocks and removing the third codon positions had no significant effect on the level of heterogeneity when comparing with data set of PCGRNA (Figure S3). The saturation analyses (Table 3) revealed that all codon positions of protein-coding genes and the mitochondrial RNA gene partitions were unsaturated for symmetric tree shapes (*Iss* < *Iss.cSym*). However, under the assumption of an asymmetrical tree, the third codon positions of protein-coding genes and the rRNA gene partition were significantly saturated (*Iss* > *Iss.cAsym*) or close to saturation. 

The averages of nonsynonymous substitution rates (*dN*) and synonymous substitution rates (*dS*) of the 55 species pairs are shown in Table 4. Our results showed that all species analyzed shared a similar *dS* value (4.45 to 4.95). In comparison, different insect groups had distinct *dN* and *dN/dS* values. Several species from Chrysopidae had the smallest *dN* (ca. 0.097) and *dN/dS* (ca. 0.021) values, while the species from Coniopterygidae (*S. aleyrodiformis* and *Coniopteryx* sp.), Dilaridae (*N. americanus* and *Dilar* sp.) and Raphidioptera (*Xanthostigma gobicola*, *Negha inflata and Inocellia fujiana*) had larger *dN* (≥ 0.162) and *dN/dS* (≥ 0.033) values than other neuropteridan species. It seemed that nonsynonymous substitution rates were correlated with the heterogeneity of sequence divergences observed in the current data.

### 3.4. Phylogenetic Analyses

**Figure 4:**
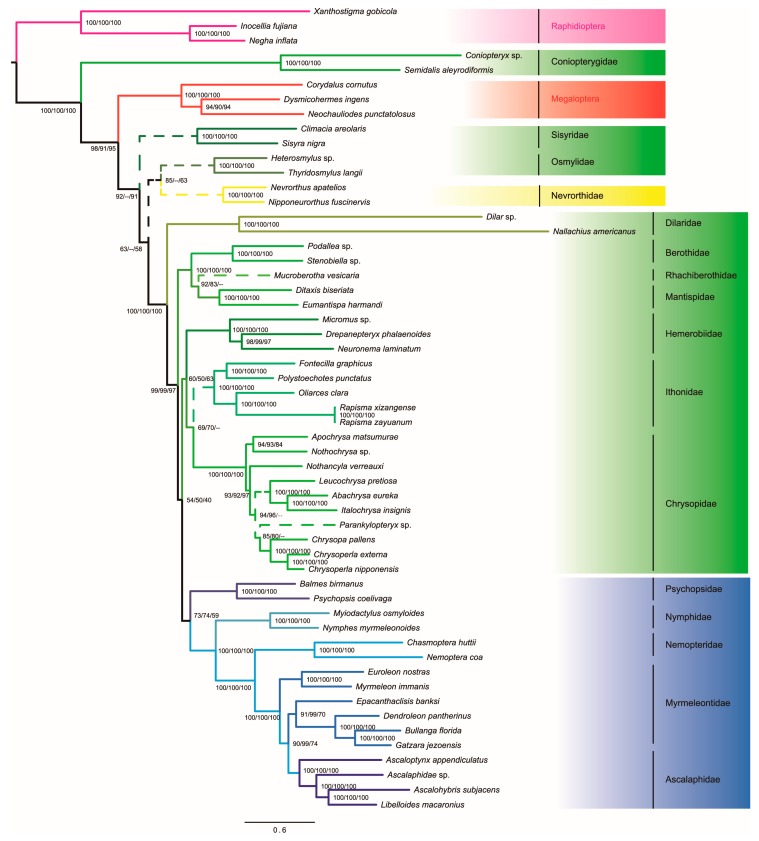
Phylogenetic tree inferred from IQ-TREE analysis of the PCGRNA data set with 55 taxa under the site-homogeneous GTR model. The clades with varying positions in the analyses of PCGRNA_Gblocks and PCG12RNA are indicated by dotted lines. Branch support values are presented near each node (Left: PCGRNA, Middle: PCGRNA_Gblocks, Right: PCG12RNA). Scale bar represents substitutions/site. The greens indicate the lineages of Hemerobiiformia and the blues indicate those of Myrmeleontiformia, while the yellow indicates the Nevrorthidae of Nevrorthiformia.

**Figure 5:**
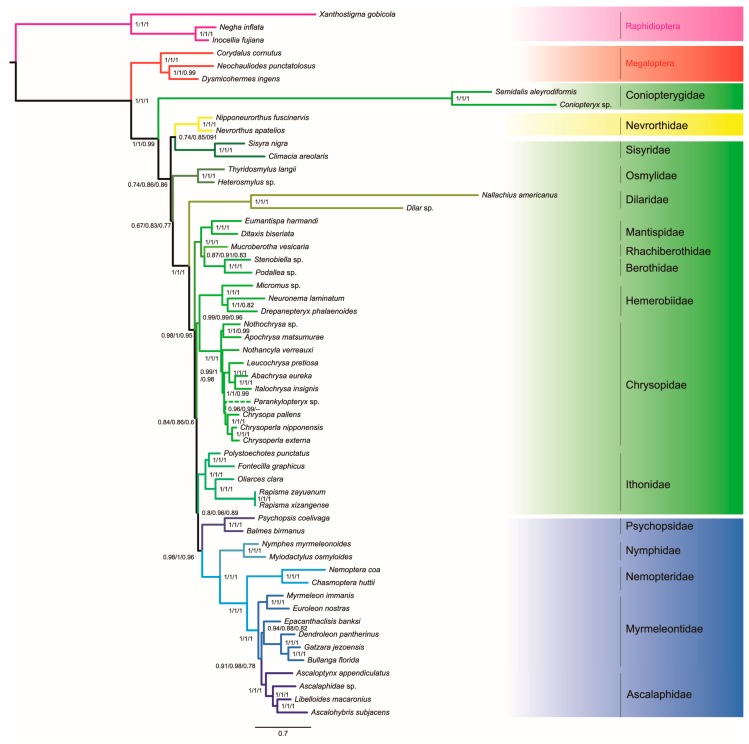
Bayesian tree inferred with PhyloBayes using the 55taxa PCGRNA data set under the site-heterogeneous CAT-GTR model. The species with different topology in the analysis from PCG12RNA is indicated by dotted lines. Values at nodes are Bayesian posterior probability support (Left: PCGRNA, Middle: PCGRNA_Gblocks, Right: PCG12RNA). Scale bar represents substitutions/site. The meaning of color is the same as those in Figure 4.

ML analyses from the data sets of PCGRNA, PCGRNA_Gblocks and PCG12RNA yielded conflicting hypotheses (Figure 4). In contrast, under the Bayesian inference analyses, data masking and removal of the third codon positions had no significant impact on the tree topology reconstruction, with only slightly changed support values at some nodes (Figure 5).

The monophyly of Neuroptera was only supported by BI analyses under the site-heterogeneous CAT-GTR model. In ML trees from the site-homogeneous GTR model, Megaloptera was nested within Neuroptera, which resulted in non-monophyly of the later. It is noteworthy that the branch lengths leading to the outgroup Raphidioptera as well as two ingroup taxa Coniopterygidae and Dilaridae are strikingly long as compared to other neuropteridan species. Long-branch attraction effect may give a reasonable explanation for the incongruence between results of ML and BI analyses. 

Myrmeleontiformia consisting of five extant families, namely Ascalaphidae, Myrmeleontidae, Nemopteridae, Nymphidae and Psychopsidae, was strongly supported by BI analyses (PP ≥ 0.96). In the ML analyses, the Myrmeleontiformia was also recovered but with low to moderate support (59 ≤ BP ≤ 74). In both ML and BI analyses, Hemerobiiformia was consistently recovered to be non-monophyletic. The monophyly of the Neuroptera families with more than two taxa included in this study was robustly supported (BP = 100, PP = 1), with the exception of Myrmeleontidae. Myrmeleontidae formed a paraphyletic grade with respect to Ascalaphidae, regardless of alignments and inference methods applied. 

Within Neuroptera, Coniopterygidae constituted the most basal clade and was sister to all other neuropteran lineages in BI analyses. The second clade included a sister-group (Nevrorthidae + Sisyridae), albeit with weak nodal support (PP ≤ 0.91). In the ML tree from PCGRNA or PCG12RNA, Sisyridae emerged as the second-earliest branching neuropteran lineage. However, in the ML tree from the PCGRNA_Gblocks, a combined clade comprising (Sisyridae + (Osmylidae + Nevrorthidae)) formed the second branching lineage of Neuroptera, albeit with low support values for deep nodes (BP ≤ 55). 

A clade including three families of Berothidae, Rhachiberothidae and Mantispidae was placed in an intermediate position within Neuroptera. However, the interrelationships among these families and support varied in different analyses. ML trees from PCGRNA and PCGRNA_Gblocks supported a branching pattern of (Berothidae + (Rhachiberothidae + Mantispidae)), whereas ML tree from PCG12RNA and all Bayesian trees found Rhachiberothidae as a sister taxon to Berothidae. The BI analysis from data set masked by Gblocks had a relatively higher node support value for the clade (Rhachiberothidae + Berothidae) than those from the data sets of PCGRNA and PCG12RNA. A sister taxon relationship between Hemerobiidae and Chrysopidae was well supported in BI analyses (PP = 0.96 or 0.99). ML analyses from PCGRNA and PCGRNA_Gblocks placed the Ithonidae as the sister group to Chrysopidae. However, the support for the position of this relationship was relatively weak (BP ≤ 70). ML tree from PCG12RNA and all Bayesian trees placed Ithonidae as the sister group to the clade Myrmeleontiformia. A stable internal relationship within the monophyletic Ithonidae was consistently retrieved by all analyses (BP = 100, PP = 1), which included two species from the Rapisma and one from the Polystoechotes.

## 4. Discussion

The triple suborder classification system of Neuroptera was proposed based mainly on the morphological characters [2,50,51]. However, molecular studies more often retrieved a paraphyletic Hemerobiiformia with regard to Myrmeleontiformia [4,5,52]. A study by Winterton et al. (2010) [14] based on morphological and molecular data also recovered Hemerobiiformia as a non-monophyletic group. The current study based on the full mitochondrial genomic data supported the non-monophyly of Hemerobiiformia in all analyses. The suborder Nevrorthiformia contained the sole family Nevrorthidae. The composition of Nevrorthidae was once regarded as the members of Sisyridae [50,53]. Zwick (1967) considered Nevrorthidae as an independent family and a sister group to the Sisyridae [54]. Both families have the truly aquatic larvae, which are distinct from other neuropteran families [6,53]. Aspöck et al. (2001) [2] placed Nevrorthidae/Nevrorthiformia to be an early offshoot and sister group to all other Neuroptera. However, the most basal placement of Nevrorthidae has been controversial [14,55,56]. A recent mitochondrial phylogenomic study also indicated a more derived position of Nevrorthidae and placed it as sister to Sisyridae [5]. A study [4] based on anchored hybrid enrichment data and including a more comprehensive taxon sampling recovered Nevrorthidae as sister to Osmylidae. In addition, Winterton et al. (2017) [57] provided morphological evidence supporting this sister relationship. Our mitochondrial genomic data recovered Nevrorthidae as a sister-group to either Osmylidae (under homogeneous model ML analyses from PCGRNA and PCG12RNA) or Sisyridae (under homogeneous model ML analysis from PCGRNA_Gblocks and under heterogeneous model BI analyses from PCGRNA, PCGRNA_Gblocks and PCG12RNA), while the placement of Nevrorthidae as sister to the rest of Neuroptera was never supported. The exact placement of Nevrorthidae needs further analysis.

In BI analyses, two representatives from Coniopterygidae exhibited remarkable long-branches compared to other neuropteran species. Coniopterygidae was recovered as the most basal group in Neuroptera (PP = 1). To investigate the potential long-branch attraction between in- and outgroups, we removed the long-branched Raphidioptera to repeat ML and Bayesian analyses. As a result, the ingroup relationships were unaffected by the reduced-outgroup set under ML analysis (Figure S4). Bayesian analysis based on the reduced-outgroup data set resulted only in slight rearrangements within basal groups of Neuroptera. The Coniopterygidae was consistently retrieved as the sister to all other neuropteran lineages in both ML and BI trees (Figures S4–S5). In previous studies based on the morphological data, the placement of Coniopterygidae was rather variable among various authors [2,7,51,56,58,59,60]. Recent molecular studies have begun to converge on the “Coniopterygidae most basal” hypothesis [4,5,14,61]. Our mitochondrial genomic data further corroborated the earliest divergence of Coniopterygidae in Neuroptera. 

Dilaridae is a small group of Neuroptera that comprises approximately 100 species in the three subfamilies [4,17]. Morphological analyses supported Dilaridae as sister group to the clade including Berothidae, Rhachiberothidae and Mantispidae [2,7,51,56,58]. All four families clustered together to form the ‘dilarid’ clade [2,7,51,55,56,58,62,63]. But morphological characters supporting the monophyly of this group is often considered to be homoplasious [4]. Recent molecular analyses provided an alternative hypothesis that Dilaridae was placed in an intermediate position between the early-branching neuropteran lineages (i.e., Coniopterygidae, Nevrorthidae, Sisyridae and Osmylidae) and the majority of Hemerobiiformia and Myrmeleontiformia [4,5]. Our analyses were in accordance with the prior molecular studies, that the Dilaridae occupied an intermediate position in the Neuroptera trees from all data sets. 

Three families of Berothidae, Rhachiberothidae and Mantispidae constitute the superfamily Mantispoidea. Fused unpaired parameres and hypermetamorphosis were proposed as the synapomorphies for the Mantispidae-Rhachiberothidae-Berothidae clade [2]. The monophyly of Mantispoidea is well resolved, although the interrelationships of taxa within this superfamily have been controversial. The Rhachiberothidae was once recognized as a member of either Mantispidae [64] or Berothidae [65]. This group is more usually considered a separate family by current authors [2,4,5,14]. Aspöck et al. (2001) [2] supported Rhachiberothidae to be sister to Berothidae based on morphological characters. The mitochondrial genome data consistently recovered Rhachiberothidae as sister to Mantispidae [5]. The anchored hybrid enrichment data retrieved Rhachiberothidae as sister to a subfamily of Mantispidae (i.e., Symphrasinae) [4]. This study gave a conflicting result on the phylogeny of Mantispoidea. ML trees from PCGRNA and PCGRNA_Gblocks strongly supported the (Berothidae + (Rhachiberothidae + Mantispidae)) clade, while ML tree from PCG12RNA and all BI trees recovered the relationships of (Mantispidae + (Rhachiberothidae + Berothidae)). Because of the better performance of the CAT-GTR model implemented in PhyloBayes in reducing systematic errors [27], the BI trees may be the most accurate reflection of Mantispoidea relationships based on the current data.

Compared with taxon sampling for the Ithonidae clade in the study of Wang et al. (2017) [5], only one additional species from *Rapisma* (i.e., *Rapisma zayuanum*) was incorporated into the current data matrix. Therefore, it is not unexpected that similar tree topology of this group is recovered. The result supported the expanded concept of the family Ithonidae to include the Rapismatidae and Polystoechotidae [3]. The Ithonidae was placed between Hemerobiidae and Chrysopidae in the ML analyses from PCGRNA and PCGRNA_Gblocks. But the support for this placement was weak (BP ≤ 69). In contrast, ML analysis from PCG12RNA and all BI analyses reconstructed Ithonidae as sister to the entire Myrmeleontiformia. The latter arrangement was more congruent with a previous mitochondrial genome study [5]. A sister-group relationship between Hemerobiidae and Chrysopidae was recovered in all BI analyses with strong support (PP ≥ 0.96). This hypothesis was also supported by the previous morphological [58] and molecular [5,52] studies.

The monophyly of Myrmeleontiformia has been well established [4,5,50,53]. The relationships among families of Myrmeleontiformia remain contentious [2,50]. Traditionally, Psychopsidae was recognized as the first diverging clade of Myrmeleontiformia, next was Nemopteridae and the Nymphidae as being sister to the (Myrmeleontidae + Ascalaphidae) clade. Aspöck (2002) [50] suggested that Psychopsidae and Nemopteridae formed a sister group and together they were sister to all other Myrmeleontiformia. The morphological analysis of Aspöck & Aspöck (2008) [58] supported Nemopteridae to be a sister-group of the (Myrmeleontidae + Ascalaphidae) clade and a sister-group relation between Psychopsidae and Nymphidae. Recent molecular studies also supported Nemopteridae as a sister-group of the (Myrmeleontidae + Ascalaphidae) clade but the sister-group relationship between Psychopsidae and Nymphidae was not returned [4,5]. In this study, each of the optimal trees indicated that the clade including Myrmeleontidae and Ascalaphidae was the sister group of Nemopteridae, followed successively by Nymphidae and Psychopsidae. This branch sequence was in agreement with the results of Wang et al. (2017) [5]. In addition, expanded taxon sampling confirmed the paraphyly of Myrmeleontidae, with respect to Ascalaphidae.

## Figures and Tables

**Table 1:** Listing of taxa included in this study. Included is the detailed taxonomic status of each species used in the phylogenetic analyses and the GenBank accession number for each species.

Item	Order	Suborder	Family	Species	Accession
					number
Ingroup	Neuroptera	Myrmeleontiformia	Ascalaphidae	*Ascalohybris subjacens*	KC758703
	Neuroptera	Myrmeleontiformia	Ascalaphidae	*Ascaloptynx appendiculatus*	FJ171324
	Neuroptera	Myrmeleontiformia	Ascalaphidae	*Libelloides macaronius*	NC_015609
	Neuroptera	Myrmeleontiformia	Ascalaphidae	*Suhpalacsa* sp.	MK301247
	Neuroptera	Hemerobiiformia	Rhachiberothidae	*Mucroberotha vesicaria*	KT425092
	Neuroptera	Hemerobiiformia	Berothidae	*Podallea sp.*	KT425091
	Neuroptera	Hemerobiiformia	Berothidae	*Stenobiella sp.*	KT425081
	Neuroptera	Hemerobiiformia	Chrysopidae	*Abachrysa eureka*	KY587199
	Neuroptera	Hemerobiiformia	Chrysopidae	*Apochrysa matsumurae*	NC_015095
	Neuroptera	Hemerobiiformia	Chrysopidae	*Chrysopa pallens*	NC_019618
	Neuroptera	Hemerobiiformia	Chrysopidae	*Chrysoperla externa*	KU877169
	Neuroptera	Hemerobiiformia	Chrysopidae	*Chrysoperla nipponensis*	NC_015093
	Neuroptera	Hemerobiiformia	Chrysopidae	*Italochrysa insignis*	KY587200
	Neuroptera	Hemerobiiformia	Chrysopidae	*Leucochrysa pretiosa*	KY587201
	Neuroptera	Hemerobiiformia	Chrysopidae	*Nothancyla verreauxi*	KP264629
	Neuroptera	Hemerobiiformia	Chrysopidae	*Nothochrysa sp.*	KP264630
	Neuroptera	Hemerobiiformia	Chrysopidae	*Parankylopteryx sp.*	KY587202
	Neuroptera	Hemerobiiformia	Coniopterygidae	*Coniopteryx sp.*	KT425078
	Neuroptera	Hemerobiiformia	Coniopterygidae	*Semidalis aleyrodiformis*	KT425067
	Neuroptera	Hemerobiiformia	Dilaridae	*Dilar sp.*	KT425073
	Neuroptera	Hemerobiiformia	Dilaridae	*Nallachius americanus*	KT425071
	Neuroptera	Hemerobiiformia	Hemerobiidae	*Drepanepteryx phalaenoides*	KT425087
	Neuroptera	Hemerobiiformia	Hemerobiidae	*Micromus sp.*	KT425075
	Neuroptera	Hemerobiiformia	Hemerobiidae	*Neuronema laminatum*	KR078257
	Neuroptera	Hemerobiiformia	Ithonidae	*Fontecilla graphicus*	KT425072
	Neuroptera	Hemerobiiformia	Ithonidae	*Oliarces clara*	KT425090
	Neuroptera	Hemerobiiformia	Ithonidae	*Polystoechotes punctatus*	FJ171325
	Neuroptera	Hemerobiiformia	Ithonidae	*Rapisma xizangense*	KF626446
	Neuroptera	Hemerobiiformia	Ithonidae	*Rapisma zayuanum*	KF626447
	Neuroptera	Hemerobiiformia	Mantispidae	*Ditaxis biseriata*	FJ859906
	Neuroptera	Hemerobiiformia	Mantispidae	*Eumantispa harmandi*	KT425080
	Neuroptera	Myrmeleontiformia	Myrmeleontidae	*Dendroleon pantherinus*	MK301246
	Neuroptera	Myrmeleontiformia	Myrmeleontidae	*Euroleon nostras*	MK301248
	Neuroptera	Myrmeleontiformia	Myrmeleontidae	*Bullanga florida*	KX369241
	Neuroptera	Myrmeleontiformia	Myrmeleontidae	*Epacanthaclisis banksi*	KF701327
	Neuroptera	Myrmeleontiformia	Myrmeleontidae	*Gatzara jezoensis*	KY364372
	Neuroptera	Myrmeleontiformia	Myrmeleontidae	*Myrmeleon immanis*	KJ461323
	Neuroptera	Myrmeleontiformia	Nemopteridae	*Chasmoptera huttii*	KT425069
	Neuroptera	Myrmeleontiformia	Nemopteridae	*Nemoptera coa*	KT425079
	Neuroptera	Nevrorthiformia	Nevrorthidae	*Nevrorthus apatelios*	KT425074
	Neuroptera	Nevrorthiformia	Nevrorthidae	*Nipponeurorthus fuscinervis*	KT425076
	Neuroptera	Myrmeleontiformia	Nymphidae	*Myiodactylus osmyloides*	KT425089
	Neuroptera	Myrmeleontiformia	Nymphidae	*Nymphes myrmeleonoides*	KJ461322
	Neuroptera	Myrmeleontiformia	Osmylidae	*Heterosmylus sp.*	KT425077
	Neuroptera	Myrmeleontiformia	Osmylidae	*Thyridosmylus langii*	KC515397
	Neuroptera	Myrmeleontiformia	Psychopsidae	*Balmes birmanus*	KT425083
	Neuroptera	Myrmeleontiformia	Psychopsidae	*Psychopsis coelivaga*	KT425082
	Neuroptera	Hemerobiiformia	Sisyridae	*Climacia areolaris*	KT425088
	Neuroptera	Hemerobiiformia	Sisyridae	*Sisyra nigra*	KT425070
Outgroup	Raphidioptera		Inocelliidae	*Inocellia fujiana*	KT425085
	Raphidioptera		Inocelliidae	*Negha inflata*	KT425086
	Raphidioptera		Raphidiidae	*Xanthostigma gobicola*	KT425093
	Megaloptera		Corydalidae	*Neochauliodes punctatolosus*	MK301249
	Megaloptera		Sialidae	*Dysmicohermes ingens*	KJ806318
	Megaloptera		Corydalidae	*Corydalus cornutus*	FJ171323

Note: Bold indicates the species newly sequenced in this study.

**Table 2:** Statistics associated to the sequencing of mitochondrial genomes using NGS-Illumina technology in four neuropteridan species.

Species	Contig Length	Mapped Bases	Mean Coverage	Total Number of Reads	Mapped Paired Reads
*Suhpalacsa* sp.	15,540	8,365,370	538	163,544,336	55,770/0.03%
*Dendroleon pantherinus*	15,416	5,518,285	357	179,658,614	36,789/0.02%
*Euroleon nostras*	9095	2,222,880	244	128,598,236	14,820/0.01%
*Neochauliodes punctatolosus*	-	-	-	-	-
*N. punctatolosus* contig-1	7013	340,174	49	128,598,236	2268/0.00%
*N. punctatolosus* contig-2	6391	607,749	95	128,598,236	4052/0.00%

**Table 3:** Substitution saturations measured by DAMBE using Xia’s method.

Gene partitions	NumOTU	*Iss^a^*	*Iss.cSym^b^*	*Psym^c^*	*Iss.cAsym^d^*	*Pasym^e^*
PCG1	32	0.349	0.808	0.000	0.554	0.000
PCG2	32	0.240	0.808	0.000	0.554	0.000
PCG3	32	0.736	0.808	0.000	0.554	0.000
PCG123	32	0.406	0.818	0.000	0.572	0.000
tRNA+rRNA	32	0.452	0.807	0.000	0.549	0.000
tRNA	32	0.361	0.773	0.000	0.488	0.000
rRNA	32	0.511	0.787	0.000	0.515	0.798

^a^*Iss*: index of substitution saturation; ^b^*Iss.cSym*: index of substitution saturation assuming a symmetrical true tree; ^c^*Psym*: probability of significant difference between *Iss* and *Iss.cSym* (two-tailed test); ^d^ *Iss.cAsym*: index of substitution saturation assuming an asymmetrical true tree; ^e^ *Pasym*: probability of significant difference between *Iss* and *Iss.cAsym* (two-tailed test).

**Table 4:** The substitution rate analyses conducted by yn00 implemented in PAML.

Species	*dN*	*dS*	*dN*/*dS*
*Abachrysa eureka*	0.101	4.517	0.022
*Apochrysa matsumurae*	0.100	4.614	0.022
*Ascalohybris subjacens*	0.130	4.758	0.027
*Ascaloptynx appendiculatus*	0.109	4.770	0.023
*Balmes birmanus*	0.099	4.621	0.021
*Bullanga florida*	0.113	4.808	0.023
*Chasmoptera huttii*	0.126	4.839	0.026
*Chrysopa pallens*	0.097	4.601	0.021
*Chrysoperla externa*	0.097	4.580	0.021
*Chrysoperla nipponensis*	0.097	4.691	0.021
*Climacia areolaris*	0.131	4.647	0.028
*Coniopteryx* sp.	0.202	4.835	0.042
*Corydalus cornutus*	0.145	4.789	0.030
*Dendroleon pantherinus*	0.111	4.780	0.023
*Dilar* sp.	0.162	4.949	0.033
*Ditaxis biseriata*	0.105	4.648	0.023
*Drepanepteryx phalaenoides*	0.114	4.688	0.024
*Dysmicohermes ingens*	0.132	4.614	0.029
*Epacanthaclisis banksi*	0.106	4.795	0.022
*Eumantispa harmandi*	0.103	4.544	0.023
*Euroleon nostras*	0.110	4.743	0.023
*Fontecilla graphicus*	0.102	4.574	0.022
*Gatzara jezoensis*	0.114	4.704	0.024
*Heterosmylus* sp.	0.125	4.598	0.027
*Inocellia fujiana*	0.173	4.584	0.038
*Italochrysa insignis*	0.106	4.594	0.023
*Leucochrysa pretiosa*	0.098	4.554	0.022
*Libelloides macaronius*	0.111	4.835	0.023
*Micromus* sp.	0.113	4.626	0.024
*Mucroberotha vesicaria*	0.100	4.626	0.022
*Myiodactylus osmyloides*	0.108	4.669	0.023
*Myrmeleon immanis*	0.105	4.733	0.022
*Nallachius americanus*	0.175	4.860	0.036
*Negha inflata*	0.174	4.570	0.038
*Nemoptera coa*	0.140	4.768	0.029
*Neochauliodes punctatolosus*	0.136	4.779	0.028
*Neuronema laminatum*	0.118	4.667	0.025
*Nevrorthus apatelios*	0.119	4.654	0.025
*Nipponeurorthus fuscinervis*	0.118	4.596	0.026
*Nothancyla verreauxi*	0.099	4.545	0.022
*Nothochrysa* sp.	0.097	4.583	0.021
*Nymphes myrmeleonoides*	0.107	4.536	0.024
*Oliarces clara*	0.106	4.624	0.023
*Parankylopteryx* sp.	0.100	4.544	0.022
*Podallea* sp.	0.113	4.696	0.024
*Polystoechotes punctatus*	0.098	4.586	0.021
*Psychopsis coelivaga*	0.102	4.704	0.022
*Rapisma xizangense*	0.120	4.452	0.027
*Rapisma zayuanum*	0.120	4.450	0.027
*Semidalis aleyrodiformis*	0.185	4.728	0.039
*Sisyra nigra*	0.131	4.600	0.028
*Stenobiella* sp.	0.108	4.626	0.023
*Suhpalacsa* sp.	0.109	4.798	0.023
*Thyridosmylus langii*	0.134	4.725	0.028
*Xanthostigma gobicola*	0.185	4.691	0.039

## Supplementary Materials

The following are available online at http://www.mdpi.com/2073-4425/10/2/108/s1, Figure S1: The secondary structures of 22 tRNA genes predicted for (A) *Dendroleon pantherinus* and (B) *Suhpalacsa* sp., Figure S2: The secondary structure of (A) *rrnL* and (B) *rrnS* genes predicted for *Suhpalacsa* sp., Figure S3: AliGROOVE heat maps of pairwise sequence comparisons for the data sets of 55taxa_PCGRNA, 55taxa_PCGRNA_Gblocks and 55taxa_PCG12RNA, Figure S4: Maximum likelihood tree inferred from the data set of 52taxa_PCGRNA using IQ-TREE under the GTR model, Figure S5: Bayesian tree inferred from the data set 52taxa_PCGRNA using PhyloBayes under the CAT-GTR model, Table S1: The best-fitting models selected by ModelFinder for the data set of (A) PCGRNA with 55 taxa, (B) PCGRNA_Gblocks with 55 taxa and (C) PCG12RNA with 55 taxa.
