# Supplementary material for: The Mitochondrial Genomes of Neuropteridan Insects and Implications for the Phylogeny of Neuroptera
Source: Genes (Basel). 2019 Feb 1;10(2):108. doi: 10.3390/genes10020108 (PMC6409651; doi:10.3390/genes10020108)
Supplement: Supplementary file 1 [file genes-10-00108-s001.pdf]

The Mitochondrial Genomes of Neuropteridan Insects and Implications for the Phylogeny of Neuroptera

Nan Song [1,*], Xin-xin Li [1], Qing Zhai [1], Hakan Bozdoğan [2] and Xin-ming Yin [1,*]

Figure S1-A. The secondary structures of the full set of tRNA genes for *Dendroleon pantherinus*.

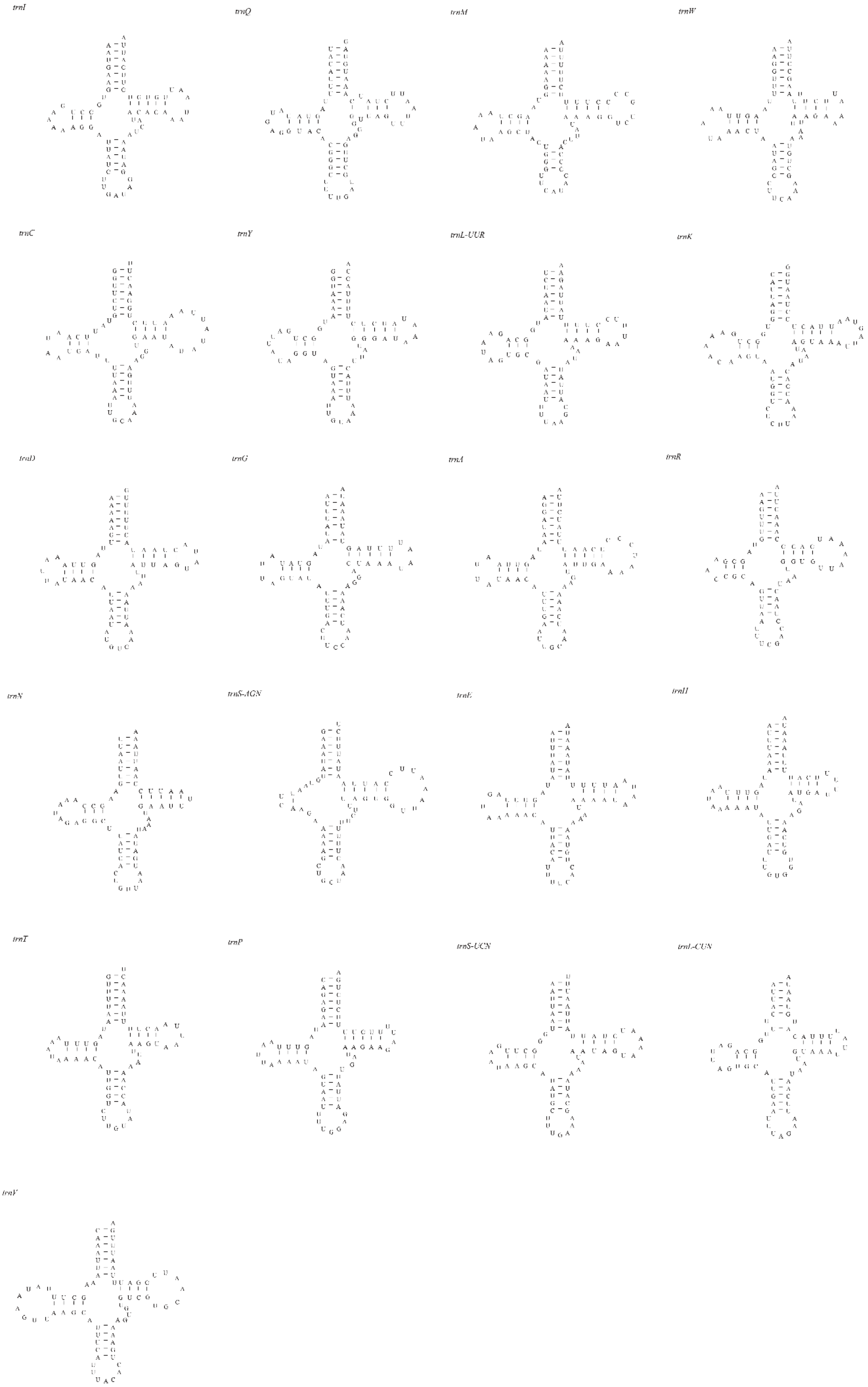

Figure S1-B. The secondary structures of the full set of tRNA genes for *Suhpalacsa* sp..

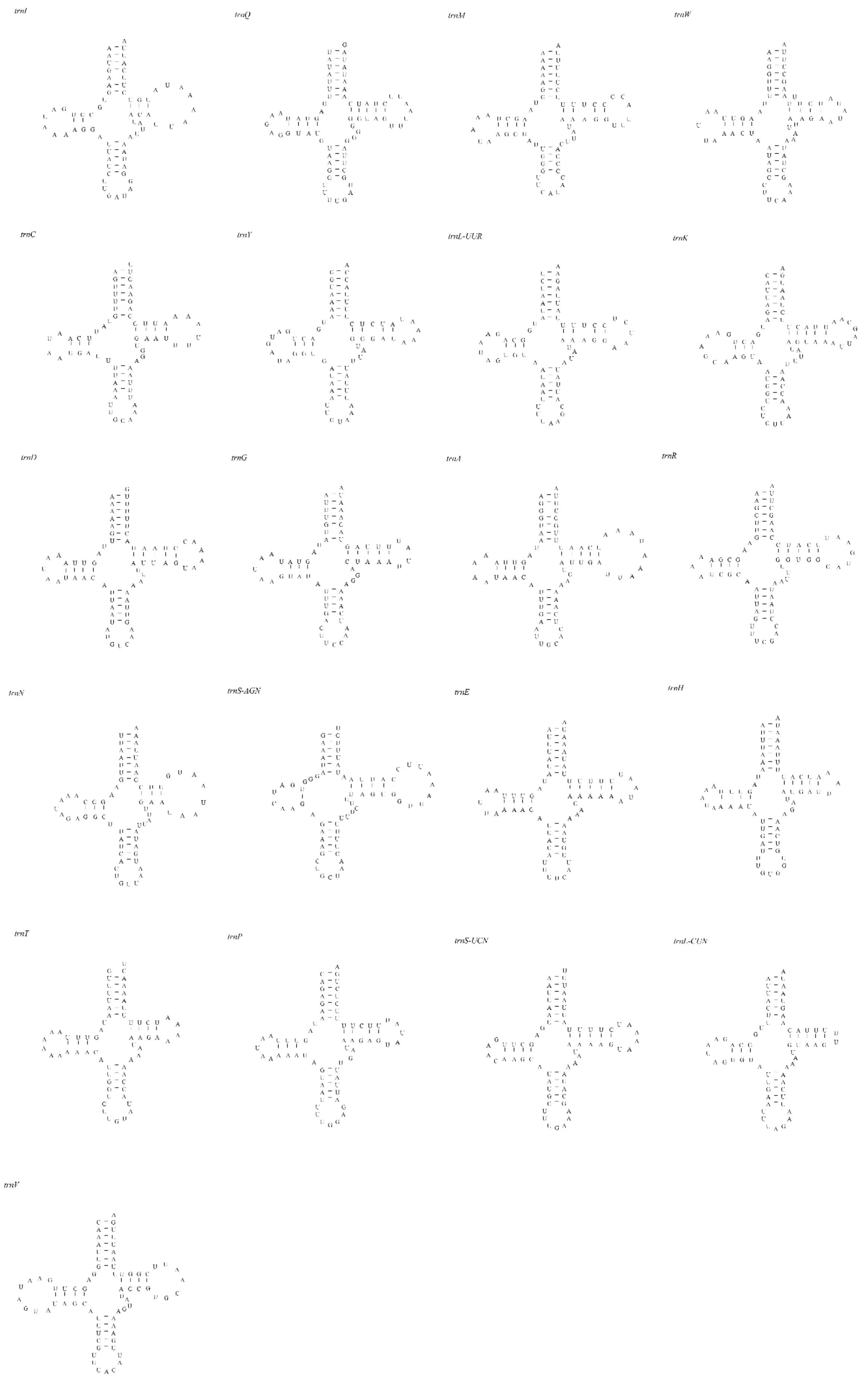

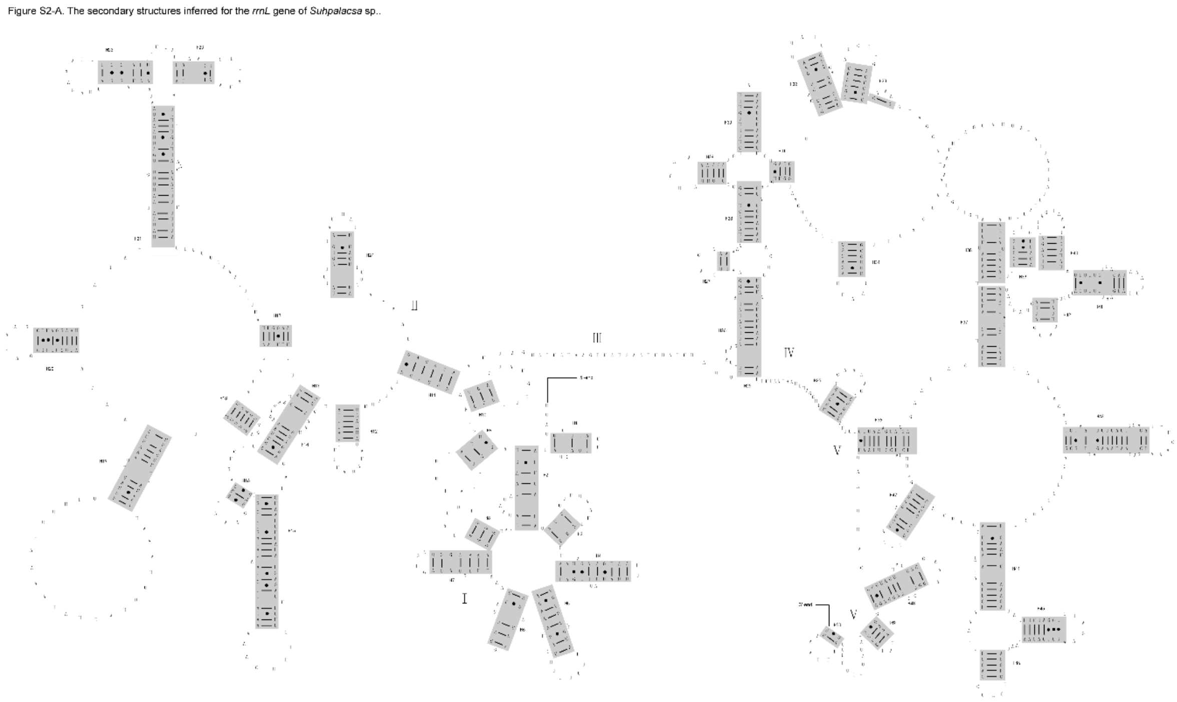

Figure S2 A. The secondary structure of *rrnL* gene predicted for *Suhpalacsa* sp..

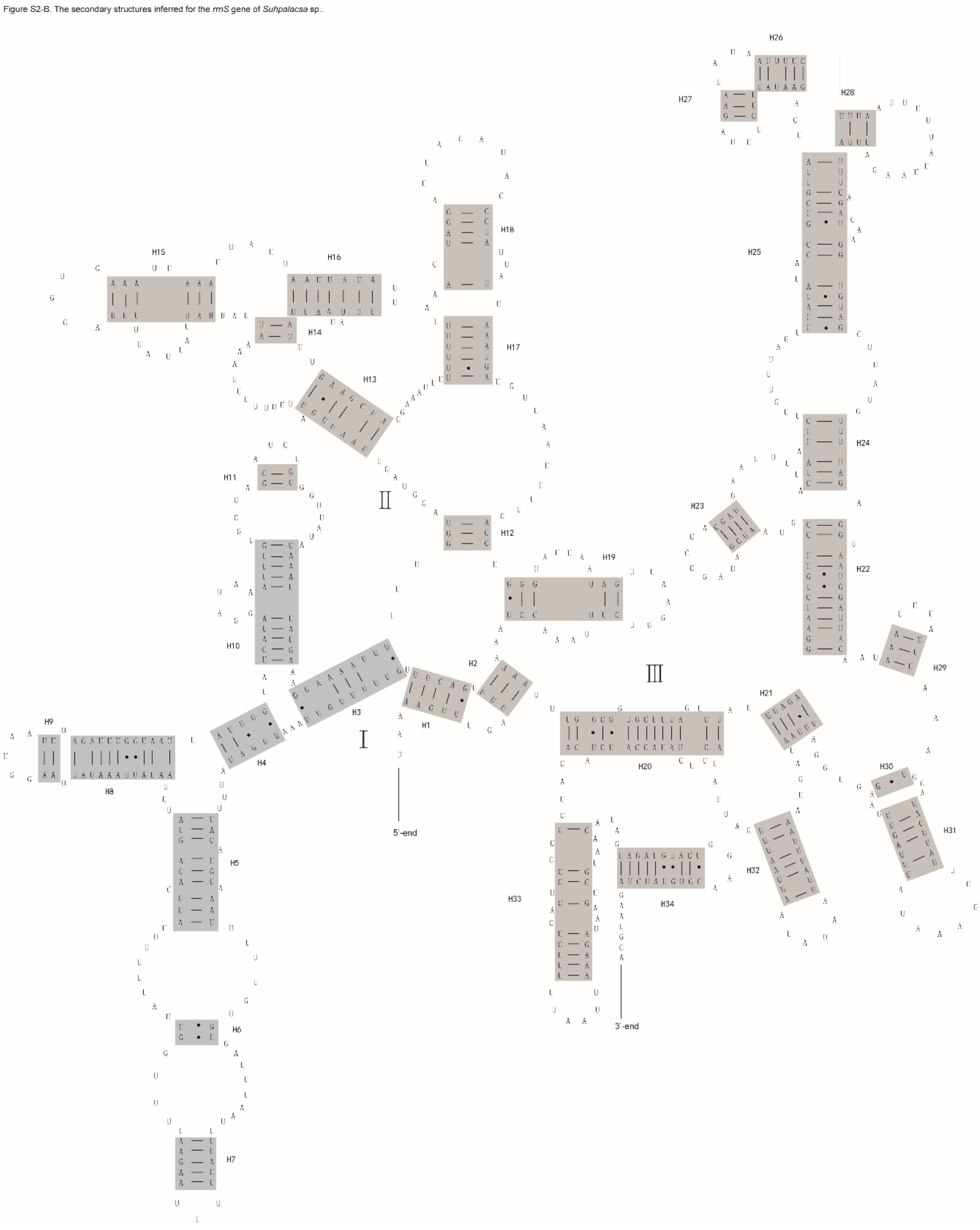

Figure S2 B. The secondary structure of *rrnS* gene predicted for *Suhpalacsa* sp..

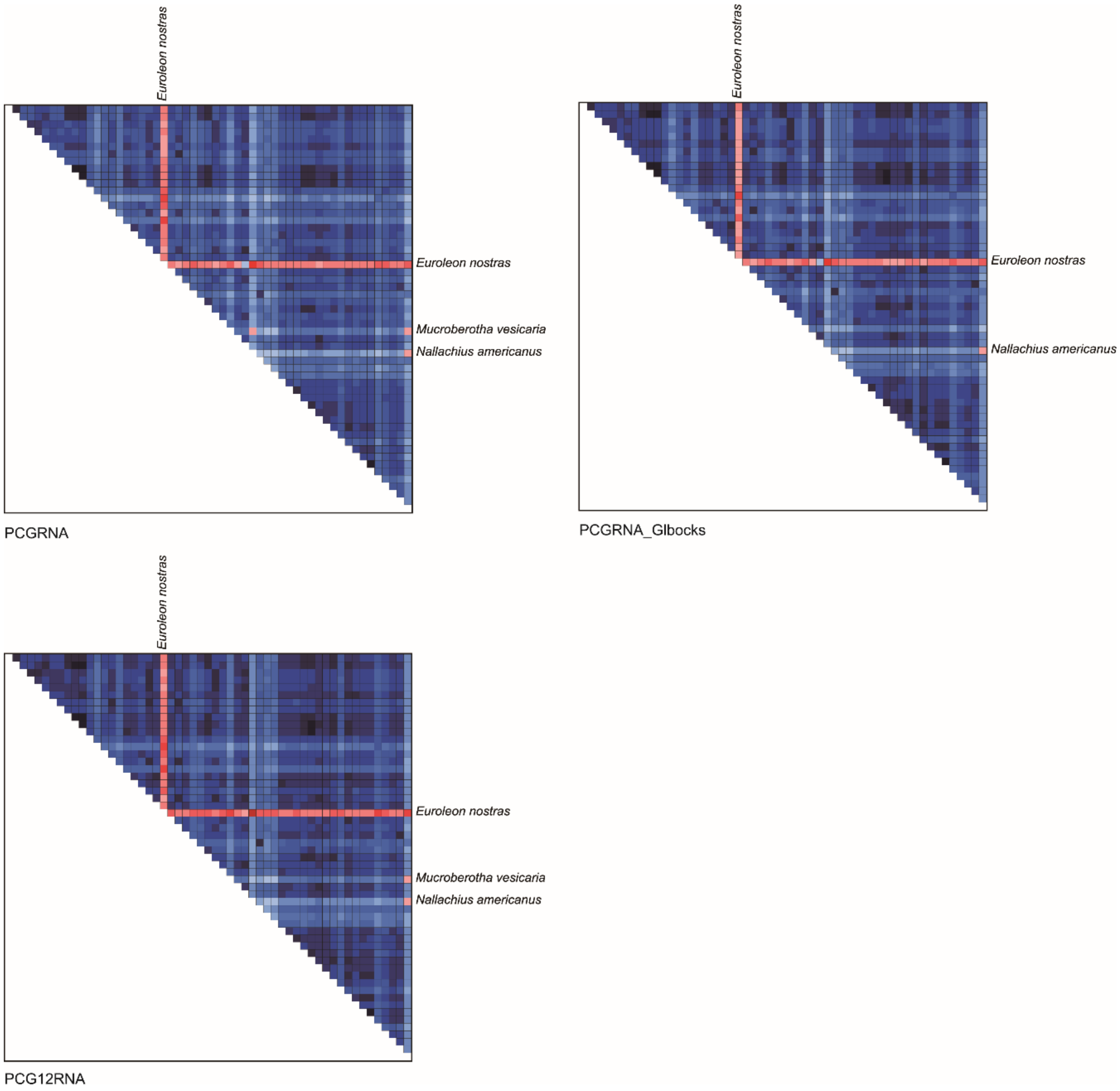

Figure S3. AliGROOVE heat maps of pairwise sequence comparisons for the data sets of 55taxa_PCGRNA, 55taxa_PCGRNA_Gblocks and 55taxa_PCG12RNA. The AliGROOVE graph shows the mean similarity scores between sequences. AliGROOVE scores range from -1 (indicating great difference in rates from the remainder of the data set, i.e. red coloring implies the significant heterogeneity) to +1 (indicating rates match all other comparisons, i.e. blue labeling).

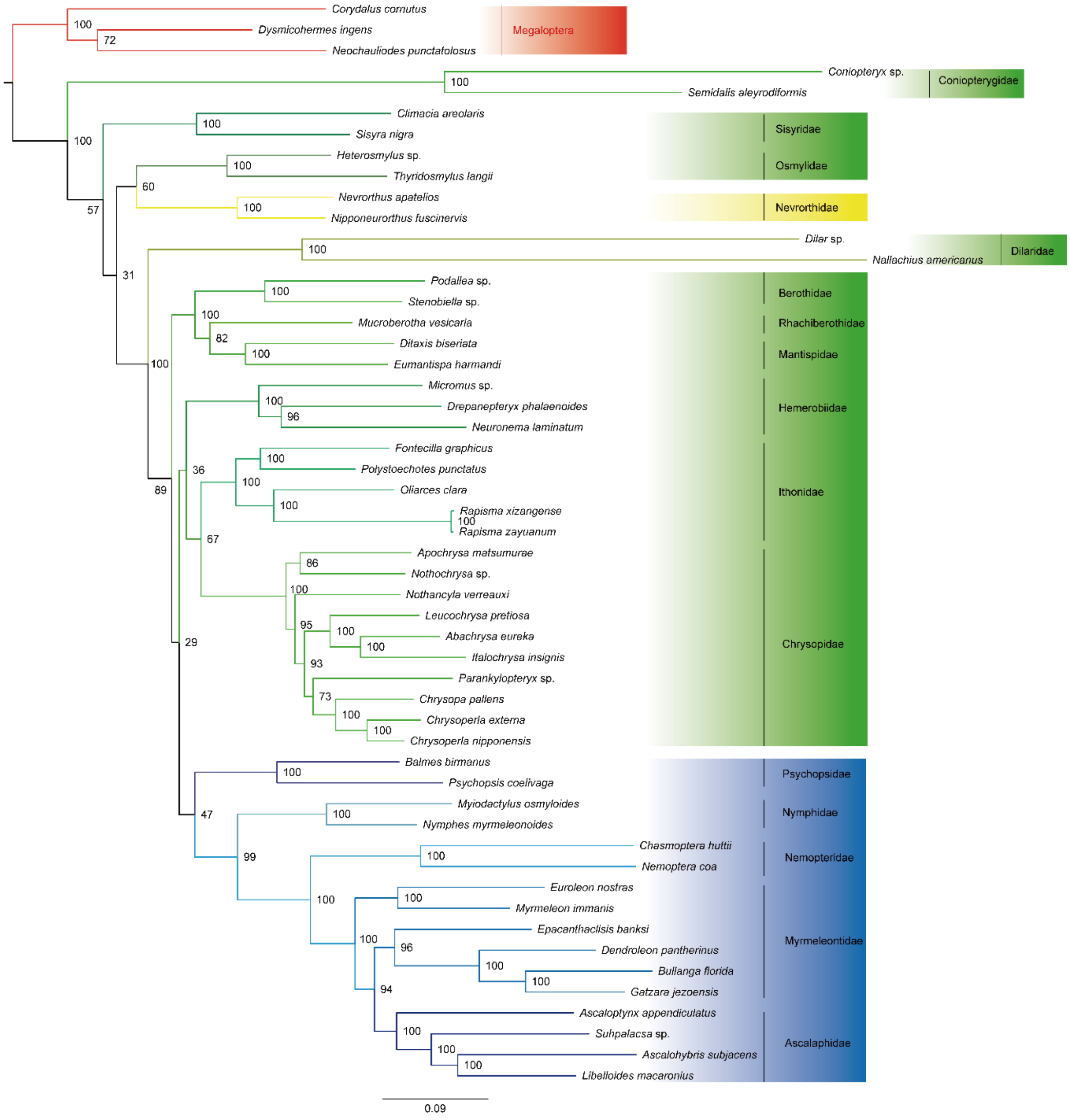

Figure S4. Maximum likelihood tree inferred from the data set of 52taxa_PCGRNA using IQ-TREE under the GTR model. Branch support values are presented near each node. Scale bar represents substitutions/site. The meaning of color is the same as those in Figure 4.

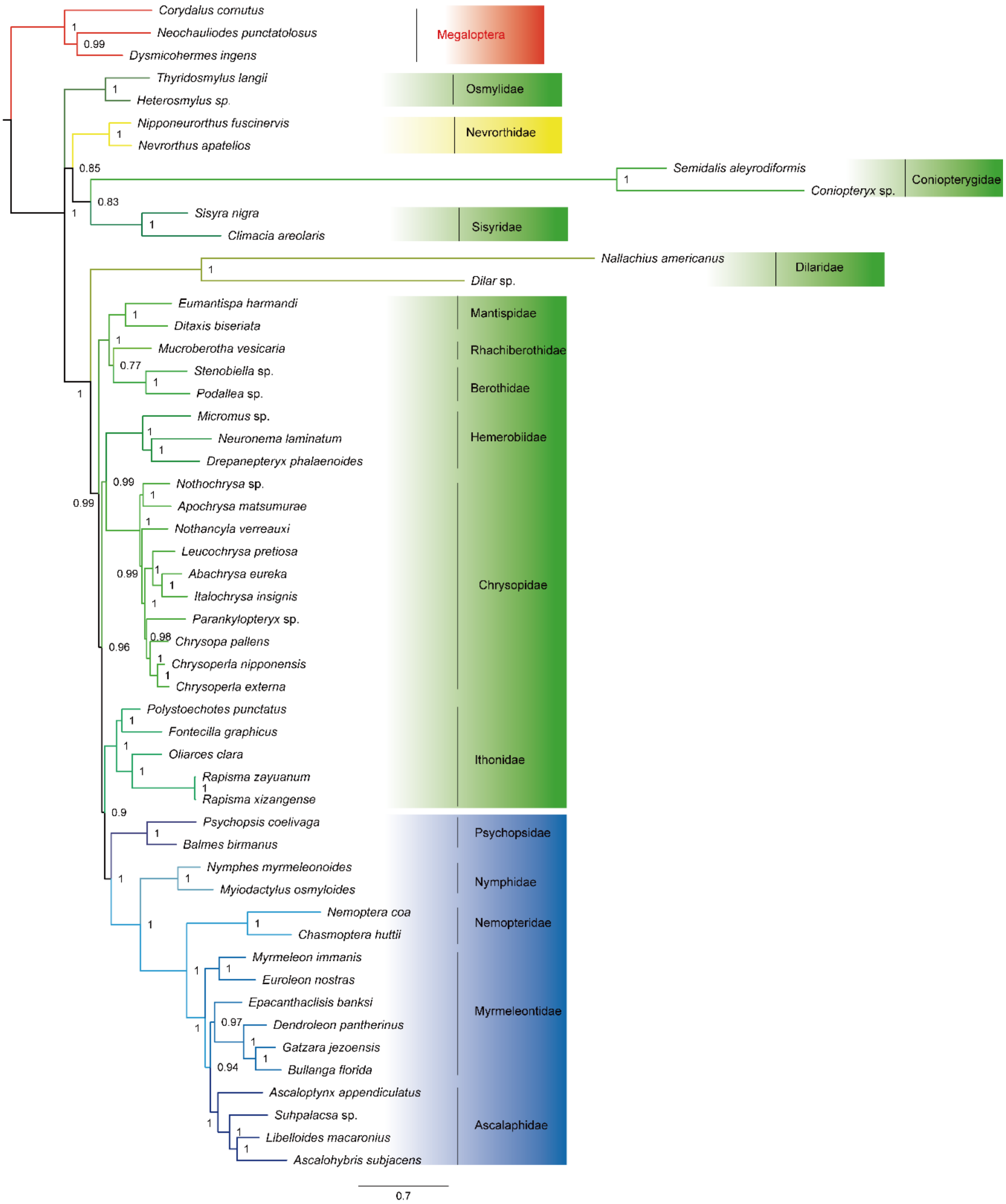

Figure S5. Bayesian tree inferred from the data set 52taxa_PCGRNA using PhyloBayes under the CAT-GTR model. Node numbers show poster probability values. Scale bar represents substitutions/site. The meaning of color is the same as those in Figure 4.

Supplementary Table S1-A. The best-fitting models selected by ModelFinder for the data set of PCGRNA with 55 taxa.

Subset	Partition names	Sites	Parsimony-informative sites	Model
part1	atp6_cp1	225	121	GTR+F+I+G4
part2	atp6_cp2	225	66	GTR+F+I+G4
part3	atp6_cp3	225	212	HKY+F+I+G4
part4	atp8_cp1	52	39	HKY+F+I+G4
part5	atp8_cp2	52	35	TPM3+F+G4
part6	atp8_cp3	52	47	TPM2u+F+G4
part7	cob_cp1	378	167	GTR+F+I+G4
part8	cob_cp2	378	90	GTR+F+I+G4
part9	cob_cp3	378	353	TN+F+I+G4
part10	cox1_cp1	512	179	GTR+F+I+G4
part11	cox1_cp2	512	71	TVM+F+I+G4
part12	cox1_cp3	512	470	TVM+F+I+G4
part13	cox2_cp1	234	116	GTR+F+I+G4
part14	cox2_cp2	234	71	GTR+F+I+G4
part15	cox2_cp3	234	210	TN+F+I+G4
part16	cox3_cp1	262	125	GTR+F+I+G4
part17	cox3_cp2	262	66	TVM+F+I+G4
part18	cox3_cp3	262	248	HKY+F+I+G4
part19	nad1_cp1	313	165	GTR+F+I+G4
part20	nad1_cp2	313	94	GTR+F+I+G4
part21	nad1_cp3	313	280	TPM3u+F+G4
part22	nad2_cp1	339	254	TIM2+F+I+G4
part23	nad2_cp2	339	181	TVM+F+I+G4
part24	nad2_cp3	339	330	TPM2u+F+G4
part25	nad3_cp1	117	71	GTR+F+G4
part26	nad3_cp2	117	45	TVM+F+I+G4
part27	nad3_cp3	117	108	TPM3+F+G4
part28	nad4_cp1	450	273	TVM+F+I+G4
part29	nad4_cp2	450	173	GTR+F+I+G4
part30	nad4_cp3	450	402	TPM3u+F+I+G4
part31	nad4l_cp1	98	67	GTR+F+I+G4
part32	nad4l_cp2	98	52	TVM+F+G4
part33	nad4l_cp3	98	88	TN+F+G4
part34	nad5_cp1	581	352	TIM2+F+I+G4
part35	nad5_cp2	581	238	GTR+F+I+G4
part36	nad5_cp3	581	527	HKY+F+G4
part37	nad6_cp1	169	143	GTR+F+G4
part38	nad6_cp2	169	102	TIM3+F+G4
part39	nad6_cp3	169	164	HKY+F+G4
part40	rrna	1892	1104	GTR+F+I+G4
part41	trna	1453	691	TVM+F+I+G4

Note: "cp_1" representing the first codon position, "cp_2" representing the second codon position, and "cp_3" representing the third codon position.

Abbreviations used in the Models selected by ModelFinder: GTR: general time reversible, variable base frequencies, symmetrical substitution matrix; HKY: Hasegawa-Kishino-Yano, variable base frequencies, one transition rate and one transversion rate; TPM: two-phase model; TN: Tamura-Nei, variable base frequencies, equal transversion rates, variable transition rates; TVM: transversion model, variable base frequencies, variable transversion rates, transition rates equal; TIM: transition model, variable base frequencies, variable transition rates, two transversion rates; F: with empirical frequencies; I: invariant sites; G: discrete Gamma distribution.

Supplementary Table S1-B. The best-fitting models selected by ModelFinder for the data set of PCGRNA_Gblocks with 55 taxa.

Subset	Partition names	Sites	Parsimony-informative sites	Model
part1	atp6	666	390	GTR+F+I+G4
part2	atp8	90	64	TVM+F+I+G4
part3	cob	1134	610	GTR+F+I+G4
part4	cox1	1533	718	GTR+F+I+G4
part5	cox2	678	388	GTR+F+I+G4
part6	cox3	777	434	GTR+F+I+G4
part7	nad1	900	506	GTR+F+I+G4
part8	nad2	876	629	GTR+F+I+G4
part9	nad3	351	224	TIM+F+I+G4
part10	nad4	1302	817	GTR+F+I+G4
part11	nad4l	270	188	GTR+F+I+G4
part12	nad5	1650	1045	GTR+F+I+G4
part13	nad6	372	280	GTR+F+I+G4
part14	rrna	1883	1095	GTR+F+I+G4
part15	trna	1443	681	TVM+F+I+G4

Supplementary Table S1-C. The best-fitting models selected by ModelFinder for the data set of PCG12RNA with 55 taxa.

Subset	Partition names	Sites	parsimony-informative sites	Model
part1	atp6	1022	402	GTR+F+I+G4
part2	atp8	196	73	GTR+F+I+G4
part3	cob	1166	312	GTR+F+I+G4
part4	cox1	662	264	GTR+F+I+G4
part5	cox2	524	191	K3Pu+F+I+G4
part6	cox3	104	53	TIM+F+I+G4
part7	nad1	756	392	TVM+F+I+G4
part8	nad2	890	508	TVM+F+I+G4
part9	nad3	336	152	K3Pu+F+I+G4
part10	nad4	452	246	GTR+F+I+G4
part11	nad4l	618	238	TIM+F+I+G4
part12	nad5	450	326	TVM+F+I+G4
part13	nad6	234	173	GTR+F+G4
part14	rrna	1892	1104	GTR+F+I+G4
part15	trna	1453	691	TVM+F+I+G4
